# Compositional Analysis of Whole Grains, Processed Grains, Grain Co-Products, and Other Carbohydrate Sources with Applicability to Pet Animal Nutrition

**DOI:** 10.3390/foods5020023

**Published:** 2016-03-25

**Authors:** Alison N. Beloshapka, Preston R. Buff, George C. Fahey, Kelly S. Swanson

**Affiliations:** 1 Department of Animal Sciences and Division of Nutritional Sciences, University of Illinois—Urbana Champaign, 1207 West Gregory Drive Urbana, IL 61801, USA; alison.beloshapka@gmail.com (A.N.B.); gcfahey@illinois.edu (G.C.F.J.); 2 The Nutro Company 1550 West McEwen Drive, Suite 100, Franklin, TN 37067, USA; Preston.Buff@effem.com

**Keywords:** amino acids, chemical composition, fiber, grains, pet food

## Abstract

Our objective was to measure the proximate, starch, amino acid, and mineral compositions of grains, grain co-products, and other carbohydrate sources with potential use in pet foods. Thirty-two samples from barley (barley flake, cut barley, ground pearled barley, malted barley, whole pearled barley, pearled barley flakes, and steamed rolled barley); oats (groats, ground oatmeal, ground steamed groats, instant oats, oat bran, oat fiber, oat flour, quick oats, regular rolled oats, steamed rolled oat groats, and steel cut groats); rice (brown rice, polished rice, defatted rice bran, and rice flour); and miscellaneous carbohydrate sources (canary grass seed, hulled millet, whole millet, quinoa, organic spelt hull pellets, potato flake, sorghum, whole wheat, and whole yellow corn) were analyzed. Crude protein, amino acid, fat, dietary fiber, resistant starch, and mineral concentrations were highly variable among the respective fractions (*i.e.*, barley flake *vs.* malted barley *vs.* steamed rolled barley) as well as among the various grains (*i.e.*, barley flake *vs.* brown rice *vs.* canary grass seed). These ingredients not only provide a readily available energy source, but also a source of dietary fiber, resistant starch, essential amino acids, and macrominerals for pet diets.

## 1. Introduction

According to the American Association of Cereal Chemists (AACC), whole grains are defined as the intact, ground, cracked, or flaked caryopsis, which consists of starchy endosperm, germ, and bran portions that are similar to the intact caryopsis, and may be malted or sprouted, as long as labeled as such [1]. Whole cereal grains belong to the *Poaceae* or *Gramineae* families, better known as grasses. The endosperm is the largest portion of the grain and contains carbohydrates, proteins, vitamins, and minerals. The germ is also known as the embryo and contains vitamins, some protein, minerals, and fats. The bran portion is the outer covering of the grain, which protects the germ and endosperm. It contains phenolic compounds, vitamins, and minerals [2]. Whole grains, processed grains, and grain components vary greatly in their macronutrient and micronutrient composition and include a variety of bioactive compounds such as fiber, folate, phenolic compounds, lignans, and sterols [3]. Whole grains have been well studied for their application to human health [2,4,5]; however, the use of whole grains or grain fractions in pet food, specifically diets intended for canines and felines, has not been thoroughly evaluated despite how significantly cereal grains are used in today’s pet food industry. These ingredients not only add nutritive value as readily available carbohydrate sources in diets for dogs or cats, but also contribute some essential vitamins, minerals, dietary fiber, fat, protein, and phytonutrients.

The largest portion of a whole grain is comprised of starch, a polysaccharide composed of many glucose units, which is linked together with α-1,4 and/or α-1,6 glycosidic linkages [6]. Some of this starch may escape digestion in the small intestine, allowing for fermentation in the colon [7], and is commonly known as resistant starch (RS). There are four main types of RS: RS1 is made up of starch that is physically entrapped within a cellular or multi-cellular structure that prevents contact with digestive enzymes (e.g., a whole grain); RS2 is made up of raw starch granules with a crystal structure resistant to digestive enzymes (e.g., raw potato starch, green banana starch, and high amylose grains); RS3 is made up of retrograded starch that forms from repeated cooking and cooling (e.g., amylose that has recrystallized into a configuration highly resistant to digestive enzymes); and RS4 is made up of chemically modified starch, including starches that have been treated with chemicals to form ether or ester linkages with starch moieties, thus inhibiting their digestion by digestive enzymes [6].

Grains are a staple carbohydrate source in both human and pet diets. While canines and felines are classified as Carnivora, the modern dog in particular has evolved to consume a more omnivorous diet, and extruded diets remain the most common diet format fed to dogs and cats [8,9,10]. Carbohydrates in the form of cereal grains or alternative sources make up anywhere from 20% to 50% of most extruded diets [11]. In comparison to animal proteins or purified fiber sources, the low cost and high nutritive value of many cereal grains make them preferred ingredients for extruded pet foods. In general, worldwide cereal grain production ranks corn (maize) as the most widely grown with the highest production, followed by rice, which is a staple to over half of the world’s population. Wheat has the third highest production, followed by barley, sorghum, and oats [12,13]. Cereal grains are widely used in the pet food industry, but poorly studied. Furthermore, many pet owners are apprehensive about feeding their pets cereal grains [14], resulting in a rise in the use of alternative carbohydrate sources, such as potatoes.

Extrusion, heat-treatment processing, and grain processing (*i.e.*, fractionation) may alter the RS content, protein quality and digestibility, and bioactive compound concentration of a grain-containing ingredient. Previous research that compared common whole grains (barley, corn, oat, rice, and wheat) and their fractions before and after processing concluded that the RS concentrations of barley, corn, oat, and wheat were decreased after processing for 1 h at 100 °C and expanded with hot air [15]. Unlike the cooking conditions used in that study which were processed in an extreme way to simulate extrusion, some whole grains are mildly processed prior to use (*i.e.*, steamed, rolled, or kilned). Roasting (at 200 °C for 8 min) and popping (at 250 °C for 90 s) of grain amaranth have been observed to decrease protein digestibility when compared to raw samples of the grain [16]. Therefore, the objective of this study was to measure the proximate, starch, amino acid (AA), and mineral composition of various whole grains, processed grains, and grain co-products that may be incorporated into pet foods and treats.

## 2. Materials and Methods

### 2.1. Samples

Thirty-two samples were analyzed (one sample of each): barley samples (barley flake, cut barley, ground pearled barley, malted barley, whole pearled barley, pearled barley flakes, and steamed rolled barley; Supplemental Figure S1); oat samples (groats, ground oatmeal, ground steamed groats, instant oats, oat bran (from two different sources), oat fiber, oat flour, quick oats, regular rolled oats, steamed rolled oat groats, and steel cut groats; Supplemental Figure S2); rice samples (brown rice, polished rice, defatted rice bran, and rice flour; Supplemental Figure S3); and miscellaneous cereals and carbohydrate sources (canary grass seed, conventional hulled millet, conventional whole millet, conventional quinoa, organic spelt hull pellets, potato flakes, sorghum, whole wheat, and whole yellow corn; Supplemental Figure S4).

### 2.2. Chemical Analyses

Grain samples were ground through a 1-mm screen in a Wiley mill (intermediate, Thomas Scientific, Swedesboro, NJ, USA). Duplicate samples were analyzed according to procedures by the Association of Official Analytical Chemists (AOAC) for dry matter (DM; 105 °C), organic matter (OM), and ash (methods 934.01, 942.05) [17]. Crude protein (CP) content was calculated from Leco total N values (model FP-2000, Leco Corporation, St. Joseph, MI, USA; method 992.15) [17]. Total lipid content (acid hydrolyzed fat; AHF) of the samples was determined according to the methods of the American Association of Cereal Chemists (AACC) [18] and Budde [19]. Gross energy (GE) of the samples was measured using an oxygen bomb calorimeter (model 1261, Parr Instruments, Moline, IL, USA). Dietary fiber concentrations [total dietary fiber (TDF), soluble dietary fiber (SDF), and insoluble dietary fiber (IDF)] were determined according to Prosky *et al.* [20]. All samples were sent to the University of Missouri Experiment Station Chemical Laboratories for AA (method 982.30E) [17] and mineral analyses, including calcium (Ca; method 985.01) [17], chloride (Cl; method 943.01) [17], magnesium (Mg; method 985.01) [17], phosphorus (P; method 985.01) [17], potassium (K; method 956.01) [17], sodium (Na; method 956.01) [17], and sulfur (S; method 956.01) [17]. Compositional data were not analyzed using statistical methods because accuracy was ensured by adequate replication, with acceptance of mean values that were within 5% of each other.

### 2.3. Starch Analyses

Grain subsamples were ground through a 0.5-mm screen in a Wiley mill (intermediate, Thomas Scientific, Swedesboro, NJ, USA). Values were determined in duplicate. The method of Muir and O’Dea [21,22] was used to determine the amount of starch digested in the stomach and small intestine by measuring glucose in the supernatant resulting from acid-enzyme digestion of the substrate. Briefly, 0.2 g of each substrate was weighed in duplicate and exposed to pepsin/HCl, amyloglucosidase, and α-amylase. Tubes containing reagents but no substrate were run as blanks. All tubes were incubated for 15 h at 37 °C and then centrifuged for 15 min. Glucose concentrations in the supernatant were determined by reading the absorbance of individual samples at 450 nm on a DU 640 spectrophotometer (Beckman Instruments, Schaumburg, IL, USA) and comparing those values against a glucose standard curve. Digestible starch (DS) was determined by subtracting (free glucose × 0.9) from (total glucose/original sample weight) present in the supernatant after 15 h of digestion. The 0.9 value used in the calculation of DS is a correction factor for the difference in weight between a free glucose (FG) unit and a glucose residue in starch. Because the measurement of glucose was used to determine starch content, the correction factor was needed. Total starch (TS) content of samples was determined using the method of Thivend *et al.* [23] with amyloglucosidase. Resistant starch was calculated by subtracting [DS + (FG × 0.9)] from TS. The released glucose value corresponds to the amount of glucose resulting from hydrolytic starch digestion that is available for absorption *in vivo*. Compositional data were not analyzed using statistical methods because accuracy was ensured by adequate replication, with acceptance of mean values that were within 5% of each other.

## 3. Results

**Figure 1:**
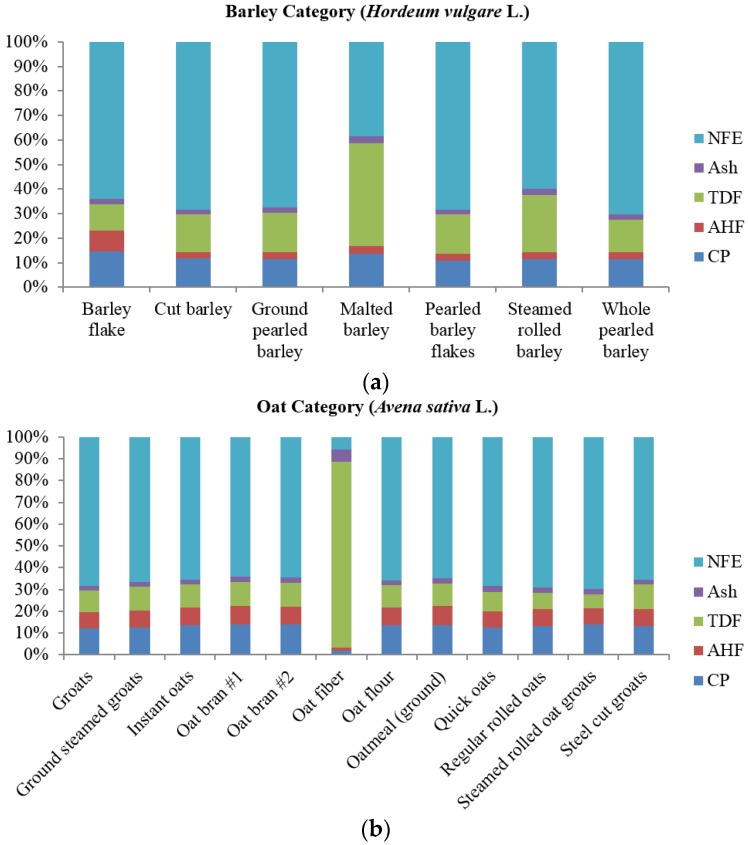

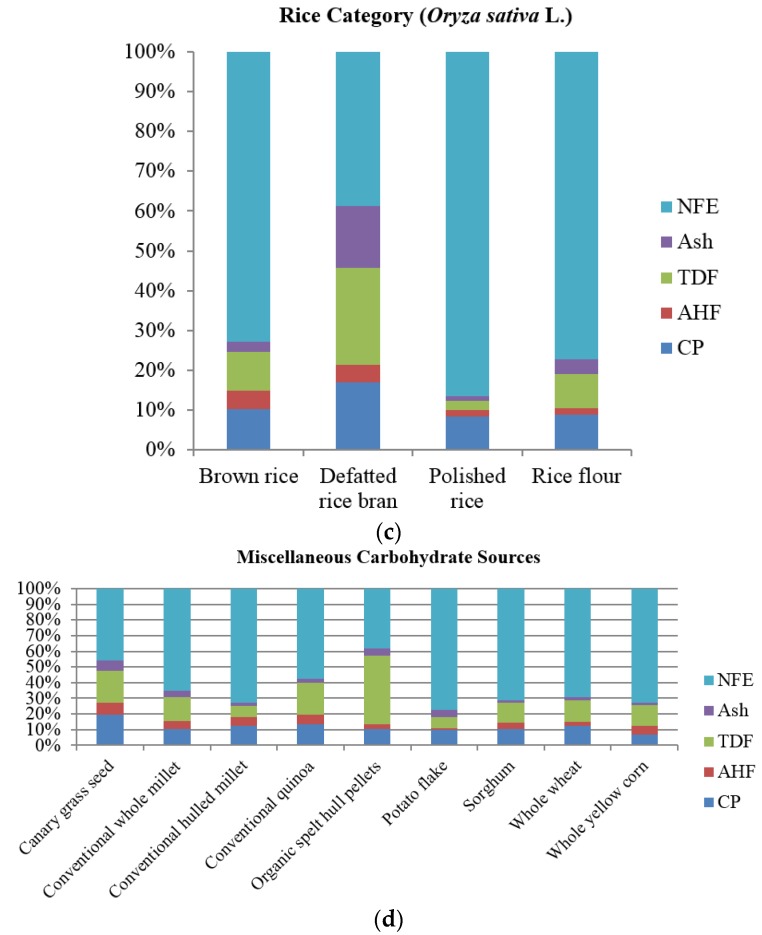
Multi-colored stacked bar graphs represent the crude protein (CP), acid hydrolyzed fat (AHF), total dietary fiber (TDF), ash, and nitrogen free extract (NFE) fractions of whole grain, processed grain, grain coproduct, and other carbohydrate sources. The ingredients are organized into 4 main categories: (**a**) barley category (*Hordeum vulgare* L.); (**b**) oat category (*Avena sativa* L.); (**c**) rice category (*Oryza sativa* L.); and (**d**) miscellaneous carbohydrate sources.

The proximate, starch, essential amino acid (EAA), non-essential amino acid, and mineral concentrations of all samples are listed in Table 1, Table 2, Table 3, Table 4 and Table 5, respectively. All values are expressed on a dry matter basis (DMB). A visual representation of the CP, AHF, TDF, ash, and nitrogen free extract (NFE) fractions of all samples are listed in Figure 1.

### 3.1. Barley Category (Hordeum vulgare L.)

Samples in the barley category had DM concentrations that ranged from 89.2% (whole pearled barley) to 95.6% (malted barley). Organic matter concentrations were very similar among all barley-based ingredients (97.3% to 98.1%). Crude protein had a greater range (10.9% to 14.5%), but was still fairly similar among ingredients. Despite the similarities in DM, OM, and CP, a couple barley-based ingredients were unique in regards to their total lipid, TDF, and starch content. Barley flake, for instance, contained 2.5 times more fat (8.5%) than any other barley-based ingredient. Total dietary fiber was quite variable as well. While most ingredients had TDF concentrations below 16%, steam rolled barley (23.1%) and malted barley (42.1%) had higher concentrations, with malted barley having approximately half of its fiber in the soluble form, which is usually the most fermentable form. Gross energy, starch, AA, and mineral concentrations were not greatly different among most barley-based ingredients. The most notable difference in terms of starch content was the malted barley, which had low total starch and the other fractions due to its higher TDF concentration.

### 3.2. Oat Category (Avenal sativa L.)

All samples in the oat category had high and similar DM (>90.4%) and OM (94.3%) concentrations. Although most oat-based ingredients had a CP and total lipid concentration above 12% and 7%, respectively, oat fiber had a very low CP (1.7%) and lipid (1.6%) concentrations. In contrast, oat fiber had a very high TDF concentration (85.2%), while all others contained less than 11%. The low lipid content led to a low gross energy content (4.44 kcal/g) of oat fiber, with all others having more than 4.7 kcal/g. Not surprisingly, oat fiber also had low starch fractions, often containing 5 to 10 times lower concentrations than the other oat-based ingredients.

### 3.3. Rice Category (Oryza sativa L.)

All samples in the rice category had high and similar DM (>88.7%) concentrations. Organic matter and CP concentrations were more variable among the rice ingredients and ranged from 84.3% to 98.7% and 8.3% to 17.0%, respectively. Gross energy concentrations were not different among the rice ingredients (~4 kcal/g) and all samples had total lipid concentrations <5%. Defatted rice bran had more TDF (24.2% TDF) than the other rice ingredients tested, most of which was insoluble (22.5% IDF). Polished rice contained the highest total starch (87.9%). All sample had similar AA concentrations. Among the rice ingredients tested, defatted rice bran was the most rich in many of the minerals tested, including Ca, P, K, and Mg.

### 3.4. Miscellaneous Cereal Grains and Other Carbohydrate Sources

All samples in the miscellaneous category had high and similar DM (>88.6%) and OM (93.8%) concentrations. Crude protein was more variable among the ingredients tested and ranged from 7.1% to 19.7%. Gross energy concentrations were not different among the miscellaneous ingredients (~4 kcal/g) and all samples had low total lipid concentrations that were all <10%. Both quinoa and canary grass seed had high TDF concentrations (20% TDF), most of which was insoluble (18% IDF). Organic spelt hull pellets also had a high TDF concentration (44% TDF) of which the majority was insoluble (39% IDF). Conventional hulled millet contained the highest total starch (73.5%) and sorghum contained the highest resistant starch (7.2%). All samples had similar AA concentrations. Potato flakes were rich in K (1.70%).

## 4. Discussion

The objective of this study was to measure the proximate, starch, AA, and mineral composition of various whole grains, processed grains, and grain co-products that may be incorporated into pet foods and treats. Cereal grains and other carbohydrate sources are widely used in pet food formulations, but poorly studied. Therefore, a detailed compositional analysis of commonly used cereal grains in addition to the *in vivo* effects of feeding novel cereal grains to pets is greatly needed. This study, which focused on the compositional analysis, may not only be useful for pet food formulators, but may be used to design *in vivo* studies to compare palatability, nutrient digestibility, and/or effects on host health in the future.

Barley (*Hordeum vulgare* L.) is rich in fermentable and soluble fibers and is gaining interest from the pet food industry as a novel carbohydrate source. Barley is harvested with the hull attached and contains a high level of β-glucans. Steamed rolled barley has steam applied above the roller mill to decrease the production of fine particles and allows for a more uniform particle size [24,25,26,27]. Cut barley, also known as barley grits, is produced when barley kernels are cut into several small pieces. If cut barley comes from hulled or hulless barley, it is considered to be a whole grain, but cut barley from pearl barley is not [25,26,27]. Barley flakes are produced when steam is applied to whole grain barley kernels, then rolled and dried. Barley flakes have decreased cooking time because of the steam that had been applied and increased the surface area [25,26,27,28]. Whole pearled barley has the hull removed and has been polished to remove some or the entire outer bran layer. Pearled barley can be tan or white and is not technically considered a whole grain, but is more nutritious than other refined grains (e.g., polished rice) due to its high concentrations of β-glucans and fiber distributed throughout the kernel. Pearled barley cooks more rapidly than whole grain barley because both the tough outer bran layer and hull have been polished off, and is the most common type of barley sold [25,27,29]. Pearled barley flakes are produced the same way as barley flakes, but from pearled barley kernels [25,26,27,28]. Ground pearled barley is pearled barley kernels that have been ground to a meal form for use as flour [25,26,27]. Malted barley requires several more processing steps: (1) initial steeping, where whole kernels are soaked to achieve a moisture content of 42%–46% (approximately 48–52 h); (2) germination, where hydrolytic enzymes are synthesized by the aleurone cells and scutellum, and are eventually secreted into the starchy endosperm of the soaked barley kernel, promoting endosperm modification (typically performed at 13–16 °C for 8–10 days); and (3) kilning, drying process to cease germination and preserve the malt (typically dried to an approximate final moisture of 2%–3%) [24]. Malted barley then can be used for brewing, distilling, or malt vinegar production [25,30]. Evaluation of the inclusion of barley into extruded dog diets is limited, but one study concluded that 40% extruded barley into a common basal diet (corn, wheat, and animal fat) resulted in decreased fecal dry matter or looser stools when fed to dogs [31]. The TDF of barley may have contributed to the looser stools observed in those dogs. The barley evaluated in the current set of ingredients had high TDF, about half of which was soluble fiber. More research is needed to fully evaluate the use of barley or barley fractions in extruded diets for dogs, but these data suggest it may be a good source of dietary fiber.

Oats (*Avena sativa* L.) are not the most common cereal grain used in pet foods, but the use of oats is growing in popularity due to their nutrient profile and lack of pet exposure, which is especially important in hypersensitivity or elimination diets [32]. Oats are harvested with the hull attached, which is often removed prior to consumption. Oat bran and germ are rarely removed, however. Like barley, oats are high in β-glucans and other constituents, such as carbohydrates, proteins, avenanthramides, tocols, lipids, alkaloids, flavonoids, saponins, and sterols [33]. Oat groats are cleaned oat kernels that have had their inedible hull removed and take the longest to cook [34]. Steel cut groats are groats that have been cut into 2–3 pieces with a sharp metal blade. They are often referred to as Irish oatmeal and cook faster than whole oat groats [35,36]. Steamed rolled oat groats are produced by applying steam to whole oat groats while being rolled into flakes [35,36]. Ground steamed groats are rolled oat groats that have been ground to a meal form [35]. Regular rolled oats are often referred to as old fashioned oats. They are created when oat groats are steamed and rolled into flakes. Regular rolled oat groats are thinner than steamed rolled oat groats, but thicker than quick or instant oats. This process also helps to preserve the healthy lipids in the oat [35,36]. Oatmeal is ground rolled oats [35,36]. Quick and instant oats are oat flakes that have been cut, then rolled thinner and steamed longer, ultimately resulting in a change in texture and decreased cooking time [35,36]. Oat bran is produced when whole oat groats are passed through several rollers that flatten the kernels. The bran is separated from the flour and sifted to be further separated, resulting in oat bran and de-branned oat flour [37]. Oat fiber is produced from finely ground oat hulls [35,36]. Oat flour is produced from whole groats sent to a stone or hammer mill that then can be used for baking or thickening of soups and stews [35,36]. Many of the aforementioned oat ingredients are readily available, cost-effective, and nutritionally consistent carbohydrate options for pet food manufacturers [32]. While not all of the health benefits identified when humans consume oats directly translate to dogs and cats, the β-glucans and dietary fiber found in oats may be beneficial in decreasing the prevalence of obesity and diabetes in pets.

Rice (*Oryza sativa* L.) is commonly used in pet foods due to its relative low cost and ease of procurement. Rice is commonly consumed worldwide and is easily digested. Rice is harvested with the hull attached and is known as paddy rice. Brown rice is a rice kernel that has had the hull removed and is rich in B vitamins and minerals [38]. Polished rice has had both the hull and bran removed, therefore making it less nutritious than brown rice [38]. Rice bran is produced from the outer layers of the harvested kernel and is rich in fiber, vitamins, and minerals [38,39], with the majority of rice bran produced being incorporated into diets for animals [40]. The high content of fiber and minerals found in rice bran may provide an alternative fiber or mineral source for canine diets. From a processing perspective, it is important to note that heat treatment can inactivate the lipase activity in the rice seed coat, greatly decreasing the shelf-life of this ingredient. This can lead to increased oxidation, rancidity, and undesirable odors and flavors [41]. Furthermore, the phytochemicals in rice bran may provide dogs and cats with additional health benefits, but these compounds were not evaluated in the current dataset. Rice flour is produced from broken white rice kernels that have been finely milled [38,42] and provides a flour option for gluten-free products.

Several cereal grains and carbohydrate sources also were evaluated, many of which are not currently used in the pet food industry. Canary grass seed (or annual canarygrass; *Phalaris canariensis* L.) is a grain crop that is produced similarly to oat and wheat and is most commonly used as birdfeed [43]. Whole yellow corn (*Zea mays* L.) is the largest size cereal grain produced and is the most produced grain worldwide, for both food and non-edible products [35]. Millet (proso variety; *Panicum miliaceum* L.) is a group of several small seeded grains. In the U.S., it is most typically used as birdfeed and livestock feed, but millet is consumed by humans in India, Africa, China, Japan, and parts of Europe [44]. Whole millet is cleaned and sized with the hull still attached, whereas hulled millet has had the hull removed. Potato flakes were first introduced as a means to increase shelf-life of potatoes. To produce potato flakes, potatoes are first peeled, trimmed, and sliced. Potato slices then are cooked at a low temperature (150–160 °C) for 20 min, known as a pre-cook step. Next, they are cooled to halt the cooking process and as a means to decrease the stickiness of the starchy vegetable. The final cooking step occurs in a steam cooker for approximately 15–60 min. Finally, the potatoes are dried in a single-drum drier and broken into flakes [45]. Quinoa (*Chenopodium quinoa* Willd.) is a tiny, round, highly nutritious pseudocereal, often light-colored, but also can be red, purple, or black [46,47]. Sorghum grain (milo; *Sorghum bicolor* L.) is a hearty grain that can survive under conditions that other grains would not (e.g., drought). Some sorghum varieties contain polyphenols or other pigments/tannins that contain anti-nutritional factors. In the U.S., it is commonly used for livestock feed, but is a great source of fiber and easily incorporated into human and pet food [48,49]. Spelt (*Triticum spelta* L.) is an ancient subspecies of wheat. Spelt is often used as an alternative feed grain, but can also be used as a food grain once the hulls are removed. Spelt hull pellets are comprised of spelt hulls that have been ground and formed into pellets [50,51]. Wheat (*Triticum aestivum* L., or bread wheat) is the most common grain used in breads, cakes and pastries, and other grain foods. Wheat contains a protein, gluten, which contributes to the elasticity of bread dough [52]. The demand for alternative carbohydrate sources is ever-growing with the increased demand for “no-corn” and “no-wheat” diets for dogs and cats. Therefore, developing a more robust database of the composition of alternative carbohydrate sources will be beneficial as the use of these alternative carbohydrates increases.

Of the whole ingredients tested (*i.e.*, whole pearled barley, groats, brown rice, canary grass seed, conventional quinoa, conventional whole millet, sorghum, whole wheat, and whole yellow corn), CP values were lowest in whole yellow corn and highest in canary grass seed. One interesting observation was the total essential amino acid concentration of quinoa, typically advertised for its high protein and essential amino acid content. However, in our evaluation, it was not one of the highest in either crude protein or essential amino acids. Fat values were lowest in whole wheat and highest in groats. Total dietary fiber concentrations were lowest in brown rice and highest in canary grass seed. Nitrogen free extract fractions were lowest in canary grass seed and highest in whole yellow corn. The demand to use less processed, more whole, natural ingredients continues to increase in both the human and pet food industries. It is possible that the use of whole grains may affect processing of the diet by contributing to increased breaking or lack of formation of the food or treat. A potential limitation of this study was the possibility that a portion of RS could have been decreased through the methodology performed to obtain the compositional data (*i.e.*, if total starch samples were allowed to cool below 55 °C, then reheated to 55 °C for 24 h, resulting in altered content of RS). This may make application or formulation using these ingredients difficult because raw ingredients were tested and it has been well documented that RS concentration and the concentrations of other nutrients can be impacted by pet food processing (e.g., extrusion; retort; baking) [15,53,54]. Furthermore, the inclusion of whole ingredients may affect the digestibility and bioavailability of nutrients in the animal, but *in vivo* testing is necessary to determine these effects.

## 5. Conclusions

In conclusion, the whole grains studied herein varied when compared to their respective fractions (*i.e.*, barley flake *vs.* malted barley *vs.* steamed rolled barley) as well as compared to other grains (*i.e.*, barley flake *vs.* brown rice *vs.* canary grass seed). The samples evaluated in this study included a variety of carbohydrate and fiber sources including: whole grains, grain fractions, processed grains, and other carbohydrate sources, such as potato flakes. Based on our analyses, we believe the most interesting ingredients for future research include oat fiber, malted barley, rice bran, canary grass seed, and barley flake. These ingredients are valuable because of their total dietary fiber content, insoluble:soluble fiber ratio, and AA profile. Compositional information generated from this study provides a framework for many grains, some of which do not have much data available. Further investigation of these ingredients for their effects *in vivo* is justified. These ingredients may not only beneficially alter indices of gastrointestinal health, but they may impact pet food formulation by potentially decreasing the amount of animal proteins needed, which can be costly from a formulation standpoint. Although grains are often called “fillers” and grain-free diets are increasingly popular with pet owners, our data demonstrate that these ingredients may contribute both as a readily available energy source and a source of dietary fiber, RS, EAA, and macrominerals for pet diets.

## Figures and Tables

**Table 1:** Chemical composition of whole grain, processed grain, grain coproduct, and other carbohydrate sources.

Item		% DM ^1^ Basis						
					Total Dietary Fiber			
	DM, %	OM	CP	AHF	TDF	IDF	SDF	GE, kcal/g DM
***Barley category (Hordeum vulgare L.)***								
Barley flake	91.8	97.8	14.5	8.5	10.8	6.5	4.3	4.8
Cut barley	89.8	98.1	11.6	2.7	15.5	10.7	4.8	4.6
Ground pearled barley	90.5	97.7	11.4	3.0	15.9	11.5	4.5	4.4
Malted barley	95.6	97.3	13.5	3.2	42.1	22.9	19.2	4.8
Pearled barley flakes	90.5	98.0	10.9	2.8	15.9	10.4	5.4	4.6
Steamed rolled barley	90.3	97.4	11.3	3.1	23.1	19.3	3.9	4.5
Whole pearled barley	89.2	97.9	11.3	2.9	13.4	8.6	4.8	4.7
***Oat category (Avena sativa L.)***								
Groats	90.5	97.6	12.1	7.4	9.9	7.9	2.0	4.7
Ground steamed groats	91.6	98.0	12.4	8.0	10.9	9.9	1.1	4.8
Instant oats	91.9	97.7	13.4	8.2	10.7	9.7	1.0	4.8
Oat bran #1	91.2	97.4	13.9	8.4	11.0	7.3	3.7	4.9
Oat bran #2	91.9	97.6	13.8	8.4	11.0	8.8	2.1	4.9
Oat fiber	95.9	94.3	1.7	1.6	85.2	80.9	4.3	4.4
Oat flour	91.9	98.0	13.6	8.1	10.4	6.0	4.4	4.8
Oatmeal (ground)	92.2	97.6	13.6	8.8	10.2	7.8	2.4	4.9
Quick oats	92.3	97.1	12.4	7.5	8.8	7.7	1.1	4.8
Regular rolled oats	91.8	97.6	13.1	8.0	7.5	6.3	1.1	4.8
Steamed rolled oat groats	90.7	97.6	13.8	7.5	6.6	3.4	3.2	4.8
Steel cut groats	90.4	97.6	13.2	7.9	11.1	8.5	2.6	4.9
***Rice category (Oryza sativa L.)***								
Brown rice	89.3	97.4	10.3	4.6	9.7	8.8	0.9	4.4
Defatted rice bran	90.6	84.3	17.0	4.4	24.2	22.5	1.7	4.3
Polished rice	88.7	98.7	8.3	1.8	2.2	1.9	0.2	4.4
Rice flour	92.4	96.1	8.9	1.5	8.6	5.6	3.0	4.1
***Miscellaneous cereal grains and other carbohydrate sources***								
Canary grass seed	93.4	93.8	19.7	7.3	20.8	17.6	3.1	4.5
Conventional whole millet	92.9	96.1	10.4	5.0	15.3	13.9	1.4	4.3
Conventional hulled millet	89.0	97.9	12.3	5.7	7.0	3.0	3.9	4.8
Conventional quinoa	92.8	97.1	13.6	6.2	19.9	18.9	1.0	4.6
Organic spelt hull pellets	89.2	95.4	10.2	3.2	44.0	39.0	5.0	4.5
Potato flake	92.0	95.5	9.7	1.3	7.0	2.8	4.2	4.3
Sorghum	88.6	98.3	10.4	4.3	12.5	9.9	2.7	4.5
Whole wheat	89.3	97.7	12.2	2.6	13.8	13.2	0.6	4.5
Whole yellow corn	88.7	98.6	7.1	5.1	13.5	12.1	1.4	4.6

^1^ DM = dry matter; OM = organic matter; CP = crude protein; AHF = acid hydrolyzed fat; TDF = total dietary fiber; IDF = insoluble dietary fiber; SDF = soluble dietary fiber; GE = gross energy.

**Table 2:** Total starch and starch fractions of whole grain, processed grain, grain coproduct, and other carbohydrate sources.

Item	% DM Basis					
	FG ^1^	TS	TS	DS	DS	RS
			(w/o FG)		(w/o FG)	
***Barley category (Hordeum vulgare L.)***						
Barley flake	0.08	67.7	67.6	63.9	63.8	3.8
Cut barley	0.13	74.2	74.1	67.0	66.9	7.2
Ground pearled barley	0.09	73.1	73.0	63.7	63.6	9.4
Malted barley	0.09	16.2	16.1	11.4	11.3	4.8
Pearled barley flakes	0.08	73.8	73.8	65.7	65.6	8.1
Steamed rolled barley	0.08	67.7	67.6	61.9	61.9	5.7
Whole pearled barley	0.11	72.3	72.2	64.9	64.8	7.4
***Oat category (Avena sativa L.)***						
Groats	0.10	73.4	73.3	69.5	69.4	3.9
Ground steamed groats	0.07	71.9	71.8	67.6	67.6	4.3
Instant oats	0.05	69.5	69.4	64.4	64.4	5.1
Oat bran #1	0.07	65.3	65.2	64.3	64.3	0.9
Oat bran #2	0.06	67.4	67.4	61.7	61.6	5.8
Oat fiber	0.08	8.5	8.5	6.9	6.8	1.7
Oat flour	0.06	69.2	69.2	65.6	65.5	3.7
Oatmeal (ground)	0.07	66.2	66.2	61.8	61.7	4.4
Quick oats	0.06	73.5	73.4	67.2	67.1	6.3
Regular rolled oats	0.05	68.7	68.7	65.0	65.0	3.7
Steamed rolled oat groats	0.06	71.2	71.1	66.1	66.0	5.1
Steel cut groats	0.06	68.4	68.3	68.2	68.2	0.1
***Rice category (Oryza sativa L.)***						
Brown rice	0.16	77.4	77.2	66.8	66.7	10.6
Defatted rice bran	0.06	34.2	34.2	29.8	29.7	4.4
Polished rice	0.06	88.0	87.9	74.7	74.7	13.2
Rice flour	0.48	73.6	73.2	66.8	66.4	6.3
***Miscellaneous cereal grains and other carbohydrate sources***						
Canary grass seed	0.17	49.7	49.5	47.3	47.1	2.4
Conventional whole millet	0.14	64.9	64.7	61.1	61.0	3.8
Conventional hulled millet	0.08	73.5	73.5	66.6	66.5	7.0
Conventional quinoa	1.31	55.7	54.5	53.3	52.2	2.4
Organic spelt hull pellets	0.16	42.0	41.9	38.3	38.2	3.7
Potato flake	0.49	73.2	72.8	66.9	66.5	6.3
Sorghum	0.20	70.5	70.4	63.3	63.1	7.2
Whole wheat	0.19	68.7	68.6	62.2	62.0	6.4
Whole yellow corn	0.24	65.0	64.8	65.6	65.3	0.0

^1^ FG = free glucose; TS = total starch; DS = digestible starch; RS = resistant starch.

**Table 3:** Essential amino acid (EAA) composition ^1^ of whole grain, processed grain, grain coproduct, and other carbohydrate sources.

Item	Essential Amino Acids (% DM Basis)													Total EAA
	Arg ^2^	His		Ile	Leu		Lys	Met		Phe	Thr	Trp	Val	
***Barley category (Hordeum vulgare*** **L.*)***														
Barley flake	0.94	0.32		0.57	1.11		0.66	0.27		0.77	0.52	0.14	0.76	6.06
Cut barley	0.51	0.23		0.41	0.80		0.42	0.19		0.61	0.39	0.11	0.56	4.23
Ground pearled barley	0.53	0.24		0.42	0.82		0.42	0.20		0.63	0.40	0.11	0.56	4.33
Malted barley	0.01	0.05		0.30	0.67		0.06	0.10		0.49	0.05	0.04	0.47	2.24
Pearled barley flakes	0.55	0.25		0.41	0.80		0.45	0.18		0.62	0.40	0.10	0.56	4.32
Steamed rolled barley	0.49	0.23		0.42	0.80		0.41	0.20		0.61	0.39	0.11	0.55	4.21
Whole pearled parley	0.58	0.25		0.42	0.82		0.48	0.20		0.62	0.42	0.10	0.59	4.48
***Oat category (Avena sativa L.)***														
Groats	0.83	0.27		0.49	0.96		0.57	0.23		0.67	0.48	0.14	0.65	5.29
Ground steamed groats	0.79	0.27		0.48	0.93		0.55	0.20		0.65	0.42	0.15	0.64	5.08
Instant oats	0.72	0.24		0.45	0.86		0.50	0.21		0.61	0.40	0.14	0.58	4.71
Oat bran #1	0.74	0.26		0.47	0.90		0.53	0.22		0.63	0.41	0.17	0.61	4.94
Oat bran #2	0.83	0.27		0.48	0.97		0.61	0.21		0.67	0.47	0.16	0.66	5.33
Oat fiber	0.86	0.28		0.49	0.97		0.64	0.22		0.68	0.48	0.17	0.66	5.45
Oat flour	0.07	0.02		0.07	0.15		0.07	0.03		0.08	0.07	<0.04	0.09	0.65
Oatmeal (ground)	0.81	0.27		0.49	0.96		0.57	0.21		0.67	0.45	0.16	0.65	5.24
Quick oats	0.69	0.24		0.43	0.84		0.51	0.18		0.60	0.39	0.15	0.56	4.59
Regular rolled oats	0.71	0.25		0.45	0.88		0.51	0.22		0.62	0.40	0.16	0.59	4.79
Steamed rolled oat groats	0.76	0.25		0.49	0.95		0.50	0.22		0.67	0.43	0.14	0.63	5.04
Steel cut groats	0.74	0.25		0.45	0.90		0.53	0.19		0.63	0.42	0.15	0.60	4.86
***Rice category (Oryza sativa L.)***														
Brown rice	0.79		0.26	0.43		0.84	0.44		0.24	0.54	0.38	0.07	0.60	4.59
Defatted rice bran	1.38		0.47	0.62		1.23	0.88		0.34	0.77	0.66	0.20	0.96	7.51
Polished rice	0.62		0.20	0.36		0.70	0.35		0.20	0.45	0.28	0.08	0.50	3.74
Rice flour	0.44		0.15	0.31		0.45	0.49		0.15	0.38	0.30	0.10	0.48	3.25
***Miscellaneous cereal grains and other carbohydrate sources***														
Canary grass seed	1.05		0.37	0.80		1.45	0.42		0.26	1.18	0.46	0.32	0.86	7.17
Conventional whole millet	1.07		0.38	0.54		0.87	0.79		0.26	0.56	0.45	0.13	0.62	5.67
Conventional hulled millet	0.40		0.24	0.51		1.49	0.20		0.36	0.69	0.39	0.08	0.61	4.97
Conventional quinoa	0.51		0.17	0.33		0.49	0.51		0.14	0.38	0.32	0.11	0.49	3.45
Organic spelt hull pellets	0.40		0.23	0.41		1.23	0.29		0.19	0.52	0.36	0.06	0.52	4.21
Potato flake	0.46		0.24	0.40		0.76	0.36		0.18	0.52	0.35	0.10	0.49	3.86
Sorghum	0.33		0.22	0.44		1.32	0.18		0.27	0.60	0.30	0.08	0.54	4.28
Whole wheat	0.55		0.27	0.44		0.84	0.40		0.20	0.57	0.39	0.18	0.54	4.38
Whole yellow corn	0.36		0.21	0.26		0.78	0.29		0.15	0.35	0.25	0.06	0.35	3.06

^1^ AOAC, 2006; method 982.30E. ^2^ Arg = arginine; His = histidine; Ile = isoleucine; Leu = leucine; Lys = lysine; Met = methionine; Phe = phenylalanine; Thr = threonine; Trp = tryptophan; Val = valine.

**Table 4:** Nonessential amino acid (NEAA) composition ^1^ of whole grain, processed grain, grain coproduct, and other carbohydrate sources.

Item	Nonessential Amino Acids (% DM Basis)												Total NEAA
	Ala ^2^	Asp	Cys	Glu	Gly	Hyl	Hyp	Orn	Pro	Ser	Tau	Tyr	
***Barley category (Hordeum vulgare L.)***													
Barley flake	0.69	1.20	0.42	3.01	0.73	0.02	0.03	0.01	0.78	0.66	0.15	0.42	8.12
Cut barley	0.45	0.68	0.23	2.71	0.43	0.03	0.01	0.00	1.24	0.45	0.17	0.26	6.66
Ground pearled Barley	0.43	0.67	0.24	2.85	0.44	0.02	0.01	0.00	1.28	0.46	0.17	0.29	6.86
Malted barley	0.42	0.46	0.03	1.97	0.33	0.05	0.02	0.06	0.86	0.02	0.14	0.24	4.60
Pearled barley flakes	0.45	0.70	0.24	2.70	0.45	0.02	0.01	0.00	1.21	0.46	0.16	0.28	6.68
Steamed rolled barley	0.42	0.64	0.24	2.81	0.43	0.02	0.00	0.00	1.28	0.45	0.17	0.24	6.70
Whole pearled barley	0.48	0.73	0.23	2.68	0.49	0.02	0.01	0.00	1.21	0.47	0.16	0.29	6.77
***Oat category (Avena sativa L.)***													
Groats	0.61	1.05	0.42	2.63	0.66	0.02	0.00	0.01	0.67	0.62	0.17	0.41	7.27
Ground steamed groats	0.57	0.95	0.38	2.57	0.63	0.01	0.01	0.01	0.64	0.56	0.17	0.37	6.87
Instant oats	0.51	0.92	0.35	2.47	0.55	0.02	0.00	0.01	0.61	0.57	0.14	0.35	6.50
Oat bran #1	0.53	0.96	0.35	2.56	0.58	0.02	0.02	0.01	0.63	0.58	0.15	0.32	6.71
Oat bran #2	0.60	1.03	0.37	2.60	0.64	0.03	0.01	0.01	0.65	0.61	0.19	0.43	7.17
Oat fiber	0.64	1.06	0.40	2.60	0.70	0.02	0.01	0.01	0.65	0.62	0.19	0.41	7.31
Oat flour	0.10	0.16	0.04	0.29	0.10	0.01	0.02	0.00	0.10	0.08	0.08	0.03	1.01
Oatmeal (ground)	0.58	1.01	0.40	2.69	0.62	0.01	0.01	0.01	0.65	0.61	0.17	0.38	7.14
Quick oats	0.50	0.90	0.36	2.41	0.54	0.01	0.01	0.01	0.60	0.55	0.15	0.29	6.33
Regular rolled oats	0.51	0.92	0.35	2.51	0.56	0.01	0.00	0.01	0.61	0.56	0.19	0.32	6.55
Steamed rolled oat groats	0.56	0.97	0.36	2.83	0.63	0.02	0.00	0.01	0.75	0.60	0.17	0.39	7.29
Steel cut groats	0.55	0.95	0.39	2.52	0.60	0.02	0.01	0.01	0.62	0.58	0.15	0.37	6.77
***Rice category (Oryza sativa L.)***													
Brown rice	0.58	0.93	0.21	1.75	0.48	0.01	0.01	0.01	0.45	0.48	0.17	0.28	5.36
Defatted rice bran	1.06	1.53	0.33	2.42	0.94	0.04	0.06	0.01	0.76	0.74	0.14	0.44	8.47
Polished rice	0.46	0.76	0.18	1.48	0.38	0.01	0.00	0.01	0.37	0.39	0.12	0.20	4.36
Rice flour	0.32	1.86	0.12	1.67	0.26	0.31	0.02	0.01	0.29	0.29	0.17	0.26	5.58
***Miscellaneous cereal grains and other carbohydrate sources***													
Canary grass seed	0.82	0.88	0.45	5.07	0.59	0.02	0.01	0.01	1.13	0.72	0.14	0.46	10.30
Conventional whole millet	0.58	1.07	0.20	1.85	0.74	0.02	0.03	0.01	0.48	0.52	0.15	0.34	5.99
Conventional hulled millet	1.26	0.74	0.20	2.54	0.33	0.01	0.01	0.00	0.82	0.70	0.16	0.27	7.04
Conventional quinoa	0.30	2.30	0.12	1.61	0.27	0.33	0.02	0.01	0.28	0.30	0.17	0.28	5.99
Organic spelt hull pellets	0.85	0.71	0.18	1.93	0.37	0.02	0.01	0.00	0.77	0.44	0.17	0.23	5.68
Potato flake	0.41	0.58	0.25	3.00	0.45	0.02	0.02	0.01	1.05	0.46	0.13	0.24	6.62
Sorghum	1.12	0.62	0.15	2.23	0.28	0.01	0.01	0.00	0.73	0.58	0.00	0.26	5.99
Whole wheat	0.45	0.66	0.27	3.50	0.50	0.01	0.01	0.01	1.20	0.57	0.17	0.27	7.62
Whole yellow corn	0.52	0.50	0.16	1.24	0.34	0.02	0.03	0.00	0.62	0.33	0.10	0.17	4.03

^1^ AOAC, 2006; method 982.30E. ^2^ Ala = alanine; Asp = aspartic acid; Cys = cysteine; Glu = glutamic acid; Gly = glycine; Hyl = hydroxylysine; Hyp = hydroxyproline; Orn = ornithine; Pro = proline; Ser = serine; Tau = taurine; Tyr = tyrosine.

**Table 5:** Mineral composition of whole grain, processed grain, grain coproduct, and other carbohydrate sources.

Item	Ca ^1,2^	Cl ^1,3^	Mg ^1,2^	P ^1,2^	K ^1,4^	Na ^1,4^	S ^1,4^
	------------------------------ % Dry Matter Basis --------------------------						
***Barley category (Hordeum vulgare L.)***							
Barley flake	0.04	<0.10	0.11	0.33	0.30	<0.10	0.10
Cut barley	0.03	<0.10	0.10	0.27	0.30	<0.10	<0.10
Ground pearled barley	0.03	<0.10	0.10	0.28	0.30	<0.10	0.10
Malted barley	0.05	<0.10	0.15	0.40	0.40	<0.10	0.10
Pearled barley flakes	0.03	<0.10	0.10	0.30	0.30	<0.10	0.10
Steamed rolled barley	0.04	<0.10	0.11	0.31	0.40	<0.10	0.10
Whole pearled barley	0.03	<0.10	0.11	0.31	0.30	<0.10	<0.10
***Oat category (Avena sativa L.)***							
Groats	0.04	<0.10	0.10	0.33	0.30	<0.10	0.10
Ground steamed groats	0.03	<0.10	0.07	0.24	0.30	<0.10	0.10
Instant oats	0.03	<0.10	0.09	0.27	0.30	<0.10	<0.10
Oat bran #1	0.03	<0.10	0.10	0.32	0.30	<0.10	0.10
Oat bran #2	0.04	<0.10	0.11	0.36	0.40	<0.10	0.10
Oat fiber	0.08	<0.10	0.06	0.04	0.50	<0.10	<0.10
Oat flour	0.04	<0.10	0.11	0.32	0.30	<0.10	0.10
Oatmeal (ground)	0.04	<0.10	0.11	0.35	0.30	<0.10	<0.10
Quick oats	0.03	<0.10	0.06	0.25	0.30	<0.10	<0.10
Regular rolled oats	0.03	<0.10	0.09	0.27	0.30	<0.10	<0.10
Steamed rolled oat groats	0.03	<0.10	0.08	0.25	0.30	<0.10	0.10
Steel cut groats	0.03	<0.10	0.08	0.25	0.30	<0.10	<0.10
***Rice category (Oryza sativa L.)***							
Brown rice	0.01	<0.10	0.13	0.37	0.30	<0.10	<0.10
Defatted rice bran	2.22	<0.10	0.88	2.03	1.50	<0.10	<0.10
Polished rice	0.00	<0.10	0.03	0.14	0.10	<0.10	<0.10
Rice flour	0.05	<0.10	0.09	0.23	1.30	<0.10	<0.10
***Miscellaneous cereal grains and other carbohydrate sources***							
Canary grass seed	0.03	<0.10	0.17	0.49	0.40	<0.10	0.20
Conventional whole millet	0.01	<0.10	0.12	0.27	0.20	<0.10	<0.10
Conventional hulled millet	0.01	<0.10	0.17	0.37	0.30	<0.10	0.10
Conventional quinoa	0.05	<0.10	0.20	0.45	0.80	<0.10	0.10
Organic spelt hull pellets	0.07	<0.10	0.15	0.40	0.40	<0.10	0.10
Potato flake	0.03	0.20	0.09	0.21	1.70	<0.10	<0.10
Sorghum	0.02	<0.10	0.18	0.36	0.40	<0.10	0.10
Whole wheat	0.03	<0.10	0.09	0.30	0.30	<0.10	<0.10
Whole yellow corn	0.00	<0.10	0.09	0.26	0.30	<0.10	<0.10

^1^ Ca = Calcium; Cl = Chloride; Mg = Magnesium; P = Phosphorus; K = Potassium; Na = Sodium; S = Sulfur. ^2^ AOAC, 2006; method 985.01. ^3^ AOAC, 2006; method 943.01. ^4^ AOAC, 2006; method 956.01.

## Supplementary Materials

Supplementary File 1
