# Supplementary material for: Compositional Analysis of Whole Grains, Processed Grains, Grain Co-Products, and Other Carbohydrate Sources with Applicability to Pet Animal Nutrition
Source: Foods. 2016 Mar 25;5(2):23. doi: 10.3390/foods5020023 (PMC5302337; doi:10.3390/foods5020023)
Supplement: Supplementary File 1 [file foods-05-00023-s001.pdf]

Supplementary Materials: Compositional Analysis of Whole Grains, Processed Grains, Grain Co-Products, and Other Carbohydrate Sources with Applicability to Pet Animal Nutrition

Alison N. Beloshapka, Preston R. Buff, George C. Fahey, Jr. and Kelly S. Swanson

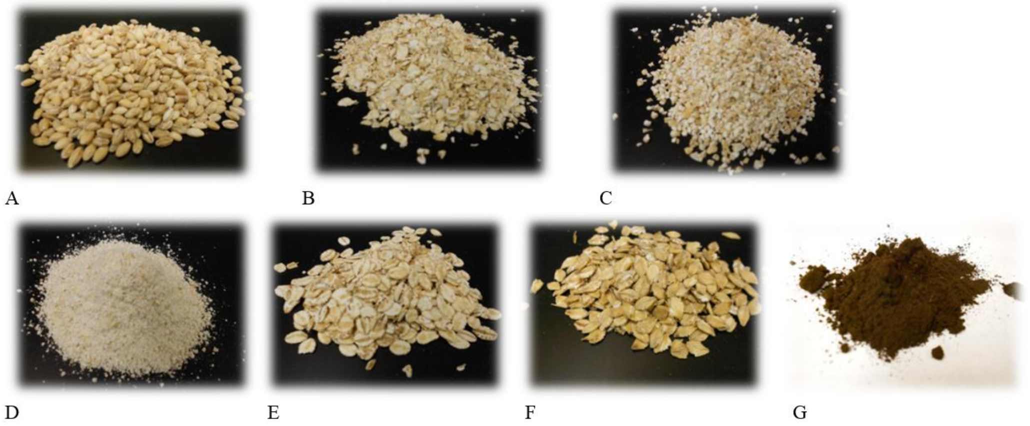

Figure S1. Photographs of barley samples before analysis. Samples included: whole pearled barley (**A**); barley flake (**B**); cut barley (**C**); ground pearled barley (**D**); pearled barley flakes (**E**); steamed rolled barley (**F**); malted barley (**G**).

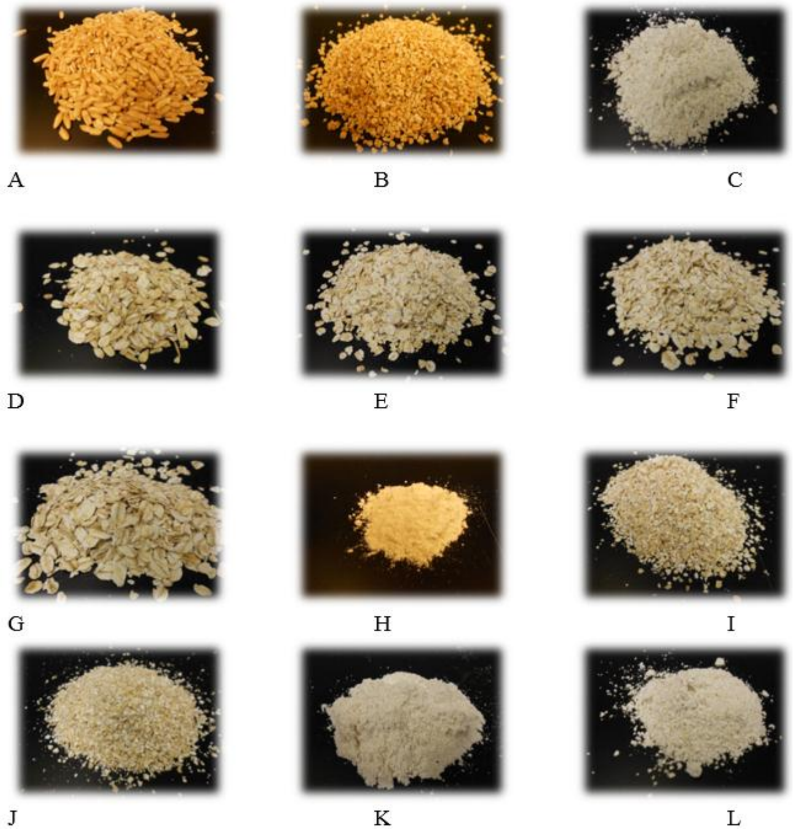

Figure S2. Photographs of oat samples before analysis. Samples included: groats (**A**); steel cut groats (**B**); ground steamed groats (**C**); steamed rolled oat groats (**D**); instant oats (**E**); quick oats (**F**); regular rolled oats (**G**); oat fiber (**H**); oat bran #1 (**I**); oat bran #2 (**J**); oat flour (**K**); oatmeal ground (**L**).

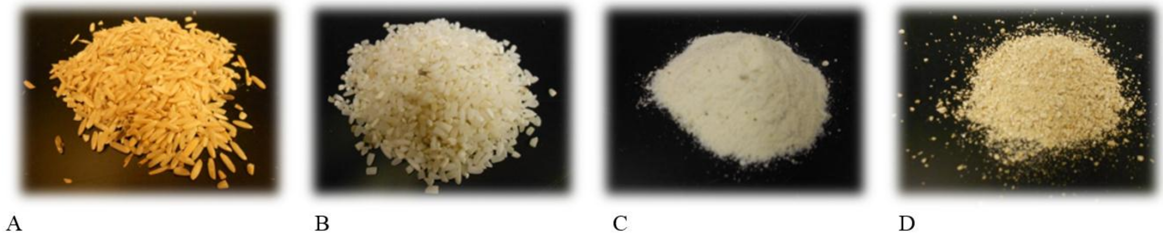

Figure S3. Photographs of rice samples before analysis. Samples included: brown rice (**A**); polished rice (**B**); rice flour (**C**); rice bran (**D**).

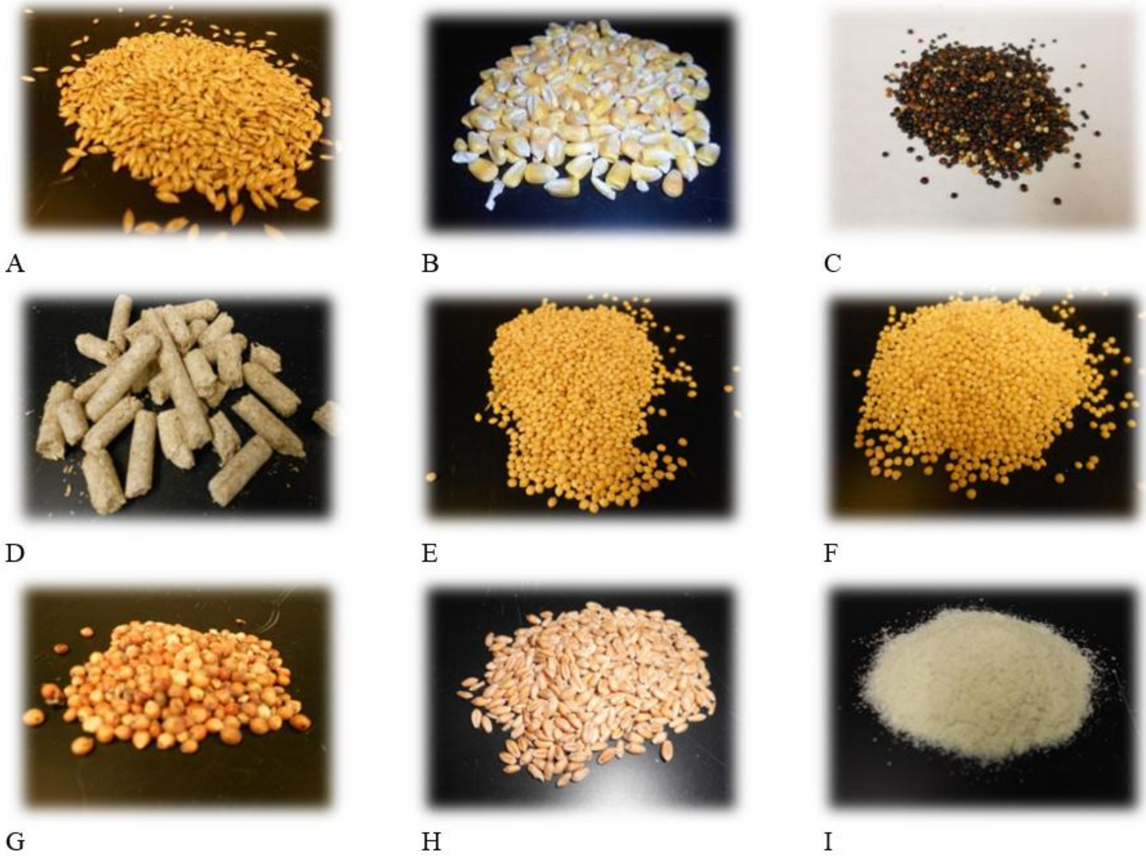

Figure S4. Photographs of miscellaneous cereals and carbohydrate sources before analysis. Samples included: canary grass seed (**A**); whole yellow corn (**B**); quinoa (**C**); organic spelt hull pellets (**D**); whole millet (**E**); hulled millet (**F**); sorghum (**G**); whole wheat (**H**); potato flake (**I**).
